# Expression of cell-surface activation markers on human CD15^+^ cells after selected non esterified fatty acid supplementations

**DOI:** 10.1186/s12944-025-02729-w

**Published:** 2025-09-29

**Authors:** A.N. Hunt, R. Cusack, M.P.W. Grocott, G. Koster, A.D. Postle, A. Dushianthan

**Affiliations:** 1 NIHR Biomedical Research Centre, University Hospital Southampton NHS Foundation Trust, Southampton, SO16 6YD UK; 2 Integrative Physiology and Critical Illness Group, Clinical and Experimental Sciences, Sir Henry Wellcome Laboratories, Faculty of Medicine, University of Southampton, Southampton, SO16 6YD UK; 3 Critical Care Research & Anaesthesia Unit, University Hospital Southampton NHS Foundation Trust, Southampton, SO16 6YD UK

**Keywords:** Neutrophils, ARDS, Phospholipids, Phosphatidylcholines

## Abstract

**Background:**

Cell surface marker (CD) expressions and membrane lipid changes characterise circulating neutrophil transmigration into inflammation sites. Specific CD expressions mandate neutrophil activation. Lipid changes alter membrane fluidity, deformability, cell spreading and transmigration. Circulating neutrophil, predominantly CD15 positive (CD15^+^), recruitment to the lungs and activation is a crucial innate immune response in Acute Respiratory Distress Syndrome (ARDS) pathology. During transmigration, their membrane lipids rapidly acquire significant whole cell arachidonic acid enrichment. The source of arachidonate is unclear but neutrophils incorporate non-esterified fatty acids (NEFA) from their microenvironment, adjusting cellular fatty acyl-CoA pools. We hypothesised that inflammation-associated, specific NEFA incorporation(s) into neutrophil membrane lipids mandate activation. We evaluated patterns of synthesis, composition and turnover of phosphatidylcholine (PC), alongside transmigration markers, following NEFA supplementations of freshly isolated human CD15^+^ cells.

**Methods:**

CD15^+^ cells were collected from venous blood samples of 5 healthy volunteers using the autoMACS system with CD15^+^ microbeads. Isolated CD15^+^ cells were incubated for 3 h with *methyl*-D_9_-choline chloride. NEFA supplements were added separately or in combination. Modified Bligh and Dyer lipid extracts were analysed by mass spectrometry. Unlabelled PC composition was determined by precursor scans of the *m/z* + 184 and the deuteriated *m/z* + 193 fragment was used to report newly synthesised *methyl*-D_9_-choline labelled PC.

**Results:**

Four NEFAs were chosen, oleic acid, linoleic acid, palmitic acid and arachidonic acid. All supplementations downregulated CD62L expression and increased expressions of CD11a and CD11b, hallmarks of activation. Membrane PC composition consisted of di-acyl species (59%) and alkylacyl PC species (41%), primary PC being PC34:1. Incorporation of *methyl*-D_9_-into polyunsaturated PC species was consistently elevated compared with endogenous composition. NEFA supplementations did not change bulk endogenous PC composition. There was lower fractional *methyl*-D_9_ enrichment with oleic acid compared to other NEFAs and the fractional enrichment varied between individual species.

**Conclusions:**

CD15^+^ microenvironmental exposure to every NEFA investigated resulted in expression of cellular activation markers. NEFA-defined changes in synthesis patterns of individual molecular species of membrane PC were consistent with membrane fluidity changes facilitating recruitment of activation markers and transmigration. Data suggest that altered neutrophil exposure to NEFA in vivo may potentially regulate their immune response.

## Introduction

Neutrophil passage, from circulation to tissue, is facilitated by a programmed sequence of specific cluster of differentiation (CD) marker expressions, plasma membrane (PM) lipid rearrangements and biophysical changes enabling altered cell deformability [1]. These changes accompany the transmigration and activation of circulating human neutrophils entering the lungs of patients with Acute Respiratory Distress Syndrome (ARDS) [2], and recent studies have highlighted a central role for neutrophil activation forming part of the pathogenesis of COVID-19 ARDS [3].

Circulating neutrophils are in constant contact with non-esterified fatty acids (NEFA) and, since they also express Free Fatty Acid Receptors FFA1 and FFA4 [4] as well as the Fatty Acid Transport Protein FATP2 [5], interaction with each NEFA may potentially modulate function, activation and fate [6]. Increases in plasma NEFA are known to accompany ARDS [7] and a significant elevation of circulating polyunsaturated NEFA in patients with severe COVID-19 pneumonia has been reported [8]. The latter study demonstrated enrichments of plasma linoleic acid (LA, 18:2, *n-6*) and arachidonic acid (AA, 20:4, *n-6*). Neutrophil transmigration in ARDS was accompanied by a four-fold increase in glycerolipid-esterified AA [9]. Consequently, studying NEFA-mediated changes in both neutrophil membrane lipids and markers of activation ex vivo may inform their potential as a target for modifying neutrophil pulmonary transmigration in both COVID-19 and other ARDS patients.

Previously, tracer NEFA radiolabelling studies using freshly isolated neutrophils in vitro [9–12] have lacked the ability, both in specificity and sensitivity, to monitor the whole membrane dynamics of their lipidome. However, the use of newer and more sensitive lipidomic molecular species profiling and analysis of bulk lipid fluxes determined from precursor labelling with stable isotopes now offers the potential of a much more detailed understanding than previously [13]. For example, a previous study from our laboratory showed that human neutrophils display a fractional synthesis of arachidonate-containing PC species that was greatly enhanced compared with composition [14]. Here we sought to model in vivo neutrophil behavior in vitro using neutrophil enriched peripheral CD15^+^ cells freshly isolated from healthy human volunteers.

We hypothesized that uptake and incorporation of specific circulating NEFA of the *n-6* series [8] may mandate neutrophil activation phenotypes. Initially we used direct supplementation with selected NEFAs together with flow cytometry to assess their capacity to alter the expression of CD62L (L-selectin), CD11a and CD11b as cell-surface markers of neutrophil activation [15]. Lipidomic methodologies were then employed to assess membrane PC composition and dynamic turnover of individual molecular species [13] over 3 h. This enabled us to probe the short-term molecular rearrangements of the patterns of newly-synthesised PC achieved following NEFA supplementation in these cells.

## Materials and methods

### Blood sampling and CD15 + cell isolation

The study part of a healthy volunteer study investigating surfactant and cellular membrane lipid metabolism and was approved by the national ethics committee 11/SC/0185) and the University Hospital Southampton Research and Development Department. Informed consent was obtained from all healthy volunteers. Venous blood samples from healthy volunteers (two male/three female) were collected in EDTA specimen sample containers. CD15 positive cells were extracted from whole blood using the autoMACS system (Miltenyi Biotec Ltd, Surrey, UK) with CD15^+^ microbeads. 1 ml of venous blood sample was mixed with 50 µl of CD15^+^ microbeads and was incubated for 15 min at 4 °C. The cells were washed with 4 ml of autoMACS running buffer (phosphate Buffered saline pH 7.2 + 2mM EDTA + 0.5% BSA + 0.05% of v/v sodium azide) and centrifuged at 445 g for 10 min at room temperature. The supernatant was discarded, and the resulting cell volume was re-suspended in 2 ml of autoMACS running buffer. The samples were then passed through a high-density magnetic cell separator and CD15^+^ cells were eluted as positive fraction. This eluted positive faction was then centrifuged at 445 g at 4 °C for 10 min. Visible cellular pellet was obtained by discarding the supernatant.

### Fatty acid incubation

Isolated CD15^+^ cells were suspended in 0.5mg *methyl*-D_9_-choline chloride (Sigma-Aldrich UK) in 1 ml of RPMI 1640 supplemented with L-glutamine and 25mM Hepes (Invitrogen, Paisley, UK). Stock solutions (3mM) of OA, LA and PA complexed with fat free albumin- were acquired commercially (Sigma-Aldrich UK), while AA-albumin (3mM) was prepared in our laboratories as previously described [16]. FA supplements were added separately or in combination at a final concentration of 30µM as indicated to each CD15^+^ cell suspension. Parallel control incubations used fat-free albumin (A8806, Sigma-Aldrich UK) at the same protein concentration. There was no control incubation without BSA, and we cannot exclude the possibility that some responses may reflect trace endotoxin contamination. Control and supplemented cells were then incubated at 37 °C for 3 h. Once the incubation was complete, the suspension was centrifuged at 445 g at 4 °C for 10 min. The incubation duration was chosen to lie within the viable life span of neutrophils ex-vivo. All CD15^+^ cell preparations were unstimulated. The resultant cellular pellets were either processed immediately for FACS analyses of CD marker expressions as described below or stored at −20 °C for lipidomic analysis.

### FACS analyses

**Fig. 1:**
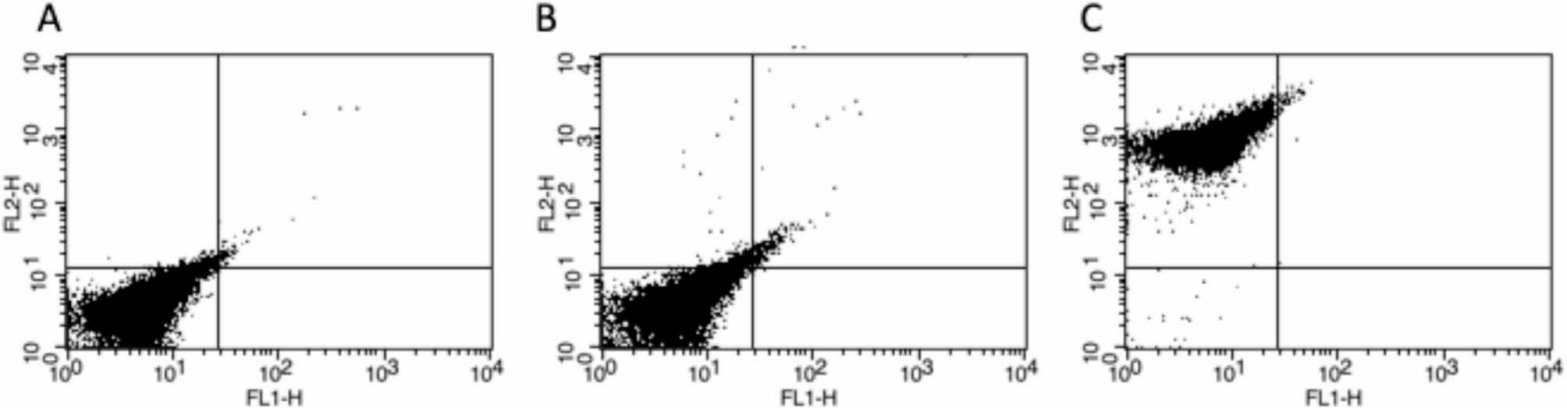
Flow cytometry analysis of CD15 + cells with (**A**) unstained, (**B**) isotype- PE control and (**C**) CD45 expression

CD15 + cells were isolated from peripheral venous whole blood. To assess the purity, isolated cells were either stained with PE-conjugated isotype control and anti-CD45-PE or unstained and analysed by flow cytometry (FACSCanto II from BD Biosciences). Propidium iodide staining was used to identify and then gate dead cells. There was no indication that NEFA supplements altered the cell viability. Cells were gated using FSC/SSC and florescence signals were recorded. Quadrant analysis was performed to assess proportion of events in that quadrant out of total events. In the control samples most cells gated in the lower left (unstained- 97%, isotype PE- 94%). When CD15 + cells stained with CD45-PE (a leukocyte marker), most (99%) gated in the upper left quadrant consistent with neutrophils or granulocytes which express CD45 (Fig. 1).

**Fig. 2:**
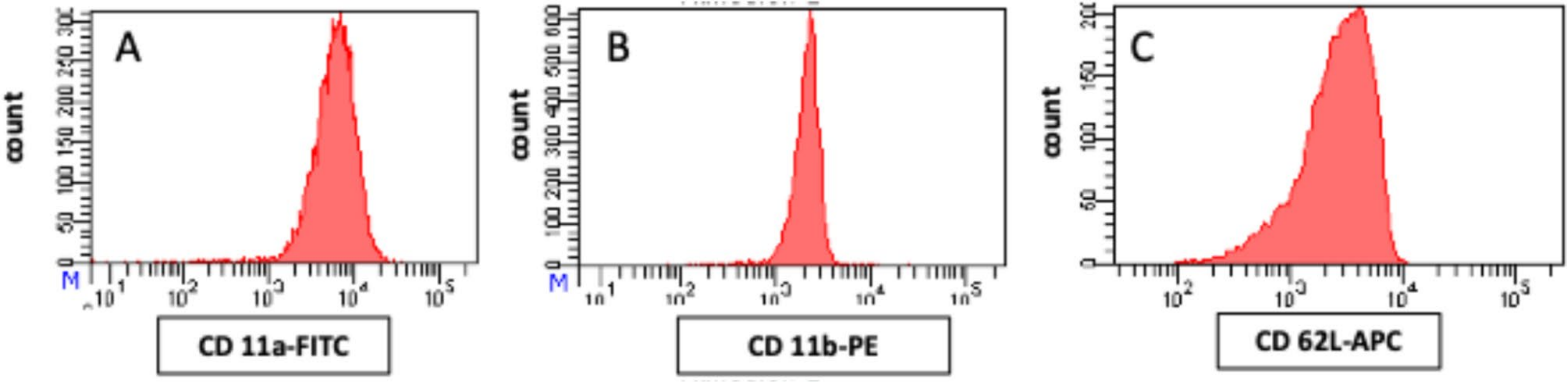
Flow cytometry histogram for fluorescence intensity for (**A**) CD11a (Integrin αL), (**B**) CD11b (Integrin αM), (**C**) CD62-L (L-selectin)

Isolated CD15 + cells were incubated individually with 5ul of anti-CD11b, anti-CD11a or anti-CD62L florescent antibodies and incubated for 15 min in dark at room temperature. The expression of CD11b, CD11a and CD62L was assessed by flow cytometry (FACSCanto II from BD Biosciences) (Fig. 2).

### Phospholipid extraction

A modified Bligh and Dyer method was applied for extraction of neutrophil PL. Samples were made up to 800 µl by the addition of normal saline. One nmole of dimyristoylphosphatidylcholine (PC14:0/14:0) was added as internal standard. Chloroform/methanol/water (2:2:1 v/v relative to the 0.8 ml) was added and then vortexed vigorously and centrifuged at 445 g for 20 min. The lower PL rich layer was carefully aspirated and dried completely at 37 °C under oxygen free N_2_ prior to analysis by nano-flow ESI-MS.

### Nano flow ESI-MS/MS analysis

The dried PL fraction was suspended in 20 µl of a mixture of methanol-butanol- water-NH_4_OH (6:2:1.6:0.4 v/v) for analysis on a Quatro Ultima triple quadrupole mass spectrometer (Micromass, Wythenshaw, UK). Samples were injected via nanoflow capillary spray to enhance intensity and specificity. The capillary voltage was set at 1.25 kV with a cone voltage of 90 V. The bulk, endogenous unlabelled PC composition was selectively determined by precursor scans of the *m/z* + 184 fragment representing the phosphocholine headgroup while precursor scans of the deuteriated *m/z* + 193 fragment were used to report newly synthesised *methyl*-D_9_-choline labelled PC as previously described [17]. PC masses were scanned between *m/z* of 670–870.

### Data extraction and analysis

Data analysis quantifying endogenous and labelled species and calculating the turnover for each PC molecular species was performed as described previously [17]. The data are presented as mean and standard error of mean. Single and multiple comparisons are made using student-tests and on-way Anova followed by Tukey HSD post hoc test respectively using SigmaPlot v16 and all relevant tests are detailed in text and figure legends as appropriate.

## Results

### Choice of specific NEFA for supplementation experiments

An intimate association between neutrophil NEFA exposure and the modulation of function, activation and fate [6], implicates uptake of circulating NEFAs as the likely primary source of the reported enrichment in AA [9, 18, 19]. So, before assessing the capacity for the activation state of neutrophils to be changed by physiologically relevant, exogenous, albumin-bound FA exposure, we first wanted to confirm that the NEFAs chosen for these experiments, in addition to AA and LA implicated in COVID-19 [8], matched the identity of major circulating NEFAs reported in the literature.

Our analyses of published population studies of healthy young adults revealed a range of circulating NEFA data, determined largely by dietary history but also in part by gender and ethnicity [20–22]. These, and many similar studies, tend to focus on the sum of both esterified and free FA rather than individual sources. However, a recent detailed lipidomic study, solely focused on albumin-bound NEFAs [21], showed four dominant FAs from over sixty FA molecular species quantified. In addition to AA at 5.7% and LA at 32.1% the two other major species were oleic Acid (OA, 18:1, *cis-9*) at 18.5% and palmitic acid (PA, 18:0) at 23.5% [21]. We elected to use these four FAs as possible manipulators of relevant neutrophil lipid pools in vivo. Importantly, we excluded NEFAs from the *n-3* series in these studies, despite their recognised role in immunomodulation, due to their lower abundance in the NEFA pool (all *n-3* FAs combined only representing around 2% of total). Also, the bulk of *n-3* FAs in the circulation are transported and presented to cells esterified on lipoprotein PL or lysoPL rather than albumin-bound [23].

### FA supplementation of CD15^+^ cells from healthy human donors and expression of markers of neutrophil maturation

We assessed the effect of single FA molecular species supplementations of CD15^+^ cells in vitro looking for recapitulation of the change of immunophenotype seen in circulating neutrophils as they are activated and migrate to the lung [2, 24]. Each albumin-bound FA, at 30µM, provided the sole exogenous lipid source and was used in separate 3 h incubations of freshly isolated of CD15^+^ cells. Post-incubation antibody labelling and FACS analysis revealed any altered surface expression of specific, functionally relevant CD markers of neutrophil activation compared with NEFA-free control incubations.

**Fig. 3:**
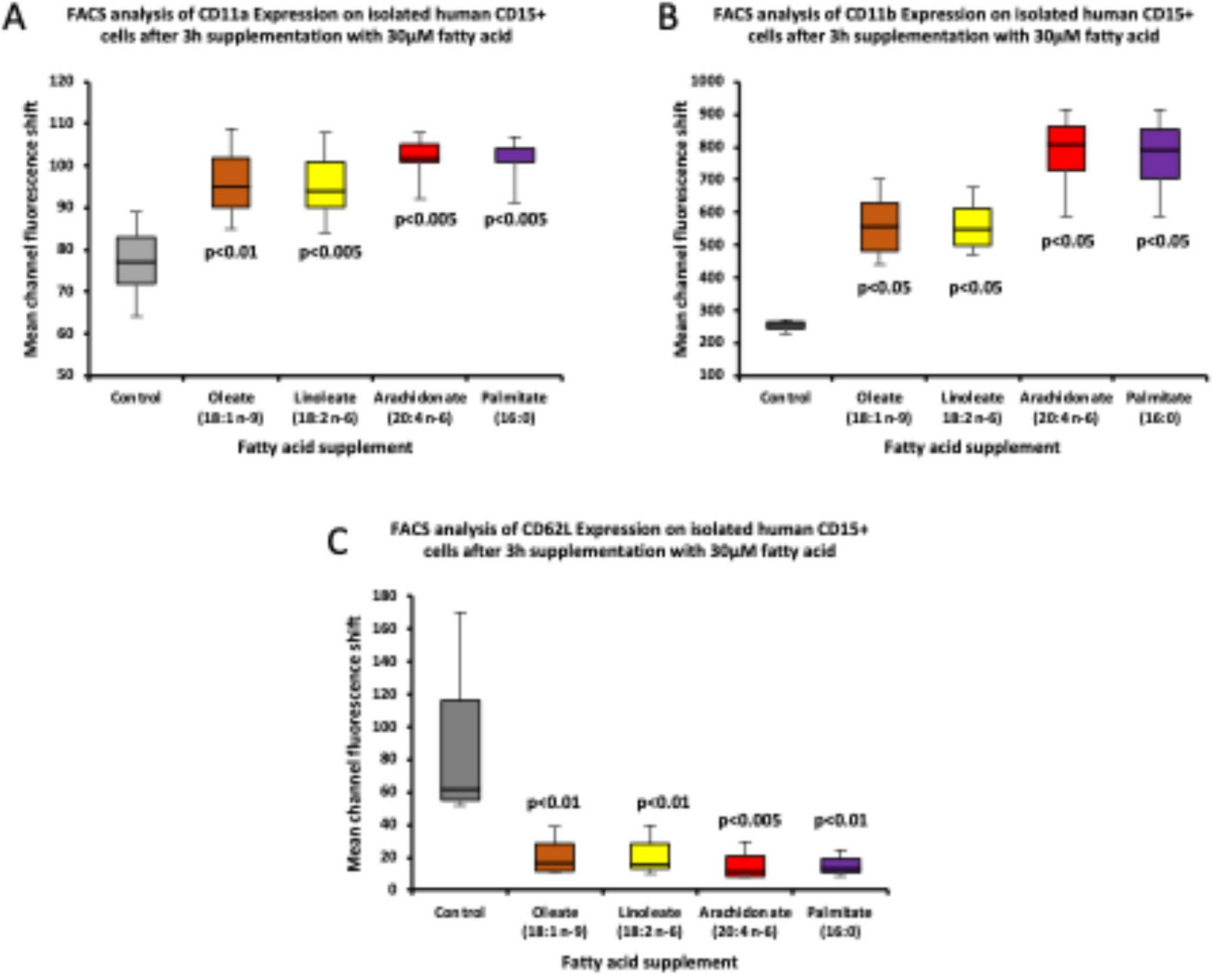
FACS Analysis for the expression of CD11a (**A**), CD11b (**B**) and CD62L (**C**) on isolated human CD15 + cells after 3 h of fatty acid (oleate, linoleate, arachidonate and palmitate) supplementation. The comparisons are made by one-way Anova followed by Tukey HSD post-hoc testing, and the p-values presented are only for the comparisons of individual fatty acid supplementation with the controls

For each NEFA evaluated, we observed significant changes in all three selected CD markers of neutrophil maturation compared with the control, non-supplemented cells from the same donor (Fig. 3). In every case, using one way ANOVA analyses, incubation with OA, LA, AA or PA resulted in (i) significantly elevated surface expression of CD11a (Fig. 3A, individual p values relative to control shown, post hoc analysis showed no significant difference between supplemented groups), (ii) significantly increased surface expression of CD11b (Fig. 3B, individual p values relative to control shown, post hoc analysis showed no significant difference between supplemented groups) and (iii) significantly decreased surface expression of CD62L (L Selectin, Fig. 3C, individual p values relative to control shown, post hoc analysis showed no significant difference between supplemented groups).

### Baseline neutrophil membrane PC composition and patterns of synthesis following 3 h incubations with and without supplemental NEFA

Early reports of the incubation of purified polymorphonuclear leucocytes with radiolabelled NEFAs, including PA, OA and LA, over a 24 h period, established uptake of all FAs, without apparent selectivity, and incorporation of label into both membrane PL and triglyceride at almost the same rate [19]. Over 80% of the PA, OA and LA that was incorporated into membrane phospholipid during supplementation studies of human neutrophils was directed to PC [25]. Surface expression of neutrophil CD markers occurs at the PM, so evaluation of any accompanying FA-induced variation in membrane PC was a logical next step. We looked specifically for changes to PC species synthesised *de novo* over 3 h incubation that might be capable of altering PM composition and potential function.

**Fig. 4:**
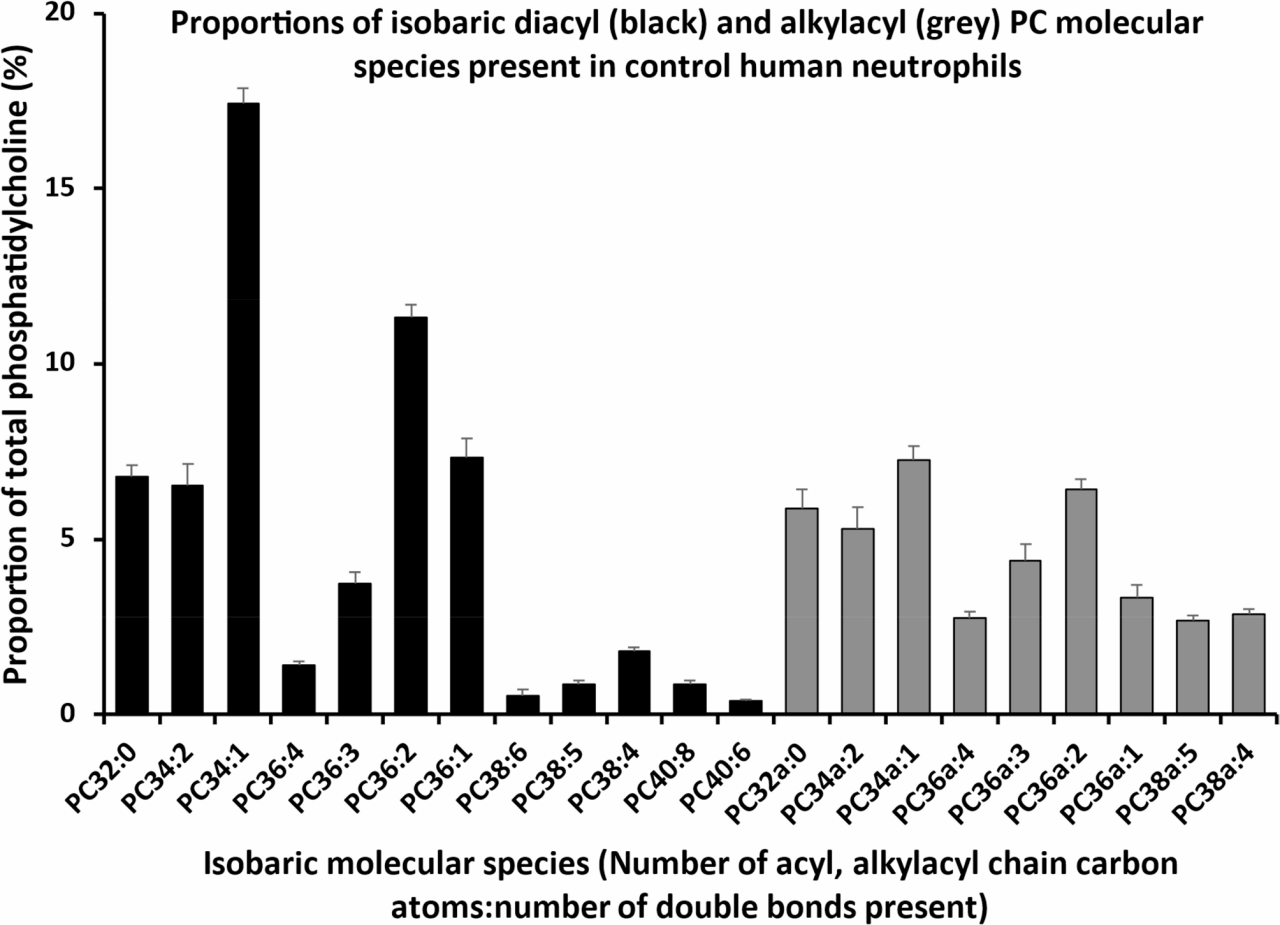
Phosphatidylcholine (PC) molecular composition of CD15 + cells. Black bars- diacyl PC species, grey bars- alkylacyl PC species

By examining the control cell data, we established normal baseline PC molecular species compositions in donor CD15^+^ cells incubated for 3 h without FA supplementation. The analyses of PC molecular species in the isobaric range *m/z* 700 – *m/z* 850 (Fig. 3) shows the distribution of membrane PC molecular species in CD15^+^ cells between diacyl PC and alkylacyl PC. At this subclass level, 59.0 ± 0.5% of total PC was represented by diacyl molecular species (Fig. 4, black bars) while alkylacyl PC species (Fig. 4, grey bars) comprised 41.0 ± 0.5% (mean ± SEM, *n* = 5) of total PC. We similarly separated diacyl from alkylacyl PC species, reflecting their differing synthesis, acylation and turnover patterns, in subsequent analyses of the data, including their 3 h PC biosynthesis profiles and the effects of fatty acid supplementations.

The diacyl PC fraction, detailed analysis showed that Mono and di-unsaturated species dominated the composition of diacyl PC at 42.6 ± 0.4% while PUFA-containing PC species with three or more double bonds, contributed a further 9.6 ± 0.5% (with a predominance of AA-rich species. In the smaller, component, Mono and di-unsaturated species were also the major alkyl-acyl PC at 22.3 ± 0.3%, but with a much greater PUFA content at 12.8 ± 0.5%, also mainly AA-rich.

### In-vitro methyl-D_9_-choline labelling of CD15 + cell PC without NEFA supplementation

**Fig. 5:**
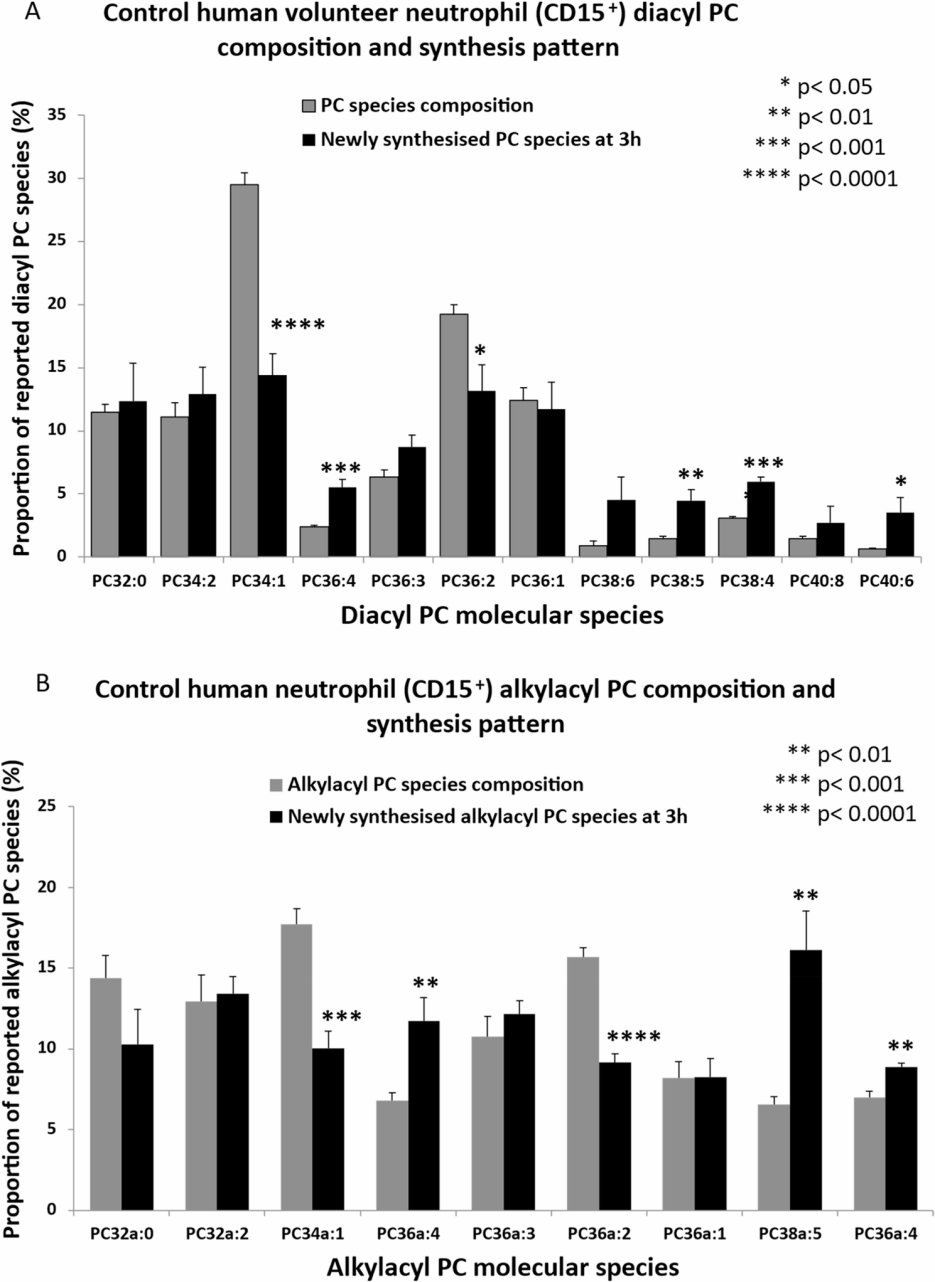
Endogenous phosphatidylcholine composition for CD15 + cells both unlabelled and newly synthesised *methyl*-D_9_-choline labelled diacyl (**A**) and alkylacyl PC (**B**) composition. The comparisons between *methyl*-D_9_ labelled and unlabelled individual species are made by using student- t- test

Inclusion of isolated CD15 + cells with the stable isotope *methyl*-D_9_ choline (at > 99% enrichment over unlabelled choline in RPMI-1640 medium) over 3 h, in the absence of other exogenous lipid, resulted in incorporation of the headgroup label into newly synthesised diacyl and alkylacyl PC species. The pattern of *methyl*-D_9_ choline labelled PC molecular species synthesised in the 3 h control incubations differed significantly from the unlabelled, bulk PC (Fig. 5A and B). This is consistent with the dynamic nature of the molecular species compositions of newly synthesised PC observed in other cells at 3 h [13, 26–28]. Within that 3 h incubation period, a large portion of newly synthesised PC is actively undergoing a remodelling as it emerges nascent on the ER and moves to its final membrane location within the cell. While the dominant newly synthesised PC species contain saturated and monounsaturated fatty acids, a significant number of additional species contain polyunsaturated species, albeit as a smaller proportion of the total. A differential turnover or remodelling of specific and relatively overabundant molecular species ultimately leads to the steady-state composition, represented here by the unlabelled bulk PC composition.

Newly-synthesises deuteriated diacyl PC (Fig. 5A) contained elevated proportions of PUFA-containing molecular species compared with bulk diacyl PC. Remodelling of these species to more saturated PCs predominantly involves acyl modification of the *sn-2* chain with the tandem sequential involvement of phospholipase A_2_ (PLA_2_) activity and specific lysoPC acyltransferase actions common to all cells including neutrophils [29, 30]. For example, the *m/z* 839 isobaric diacyl PC40:8 species identified here corresponds with the *m/z* 830 isobaric equivalent in the unlabelled PC and is overabundant with respect to the steady state. Fragmentation of the peak showed that it contains a majority proportion of diarachidonyl species (20:4/20:4) implying remodelling also occurs at the *sn-1* position, consistent with a sequential phospholipase A_1_ (PLA_1_)/acyltransferase activity.

Two alkylacyl *methy*l-D_9_-choline containing PC species (Fig. 5B) PUFA species were significantly enriched, PC36a:4 and PC38a:5. The PUFA content of two species was predominately AA, the overabundance of which was not retained in unlabelled alkylacyl species at steady state and was consistent with *sn-2* PUFA turnover/remodelling.

### Methyl-D_9_-choline labelling of CD15 + cell PC with NEFA supplementation in vitro

**Fig. 6:**
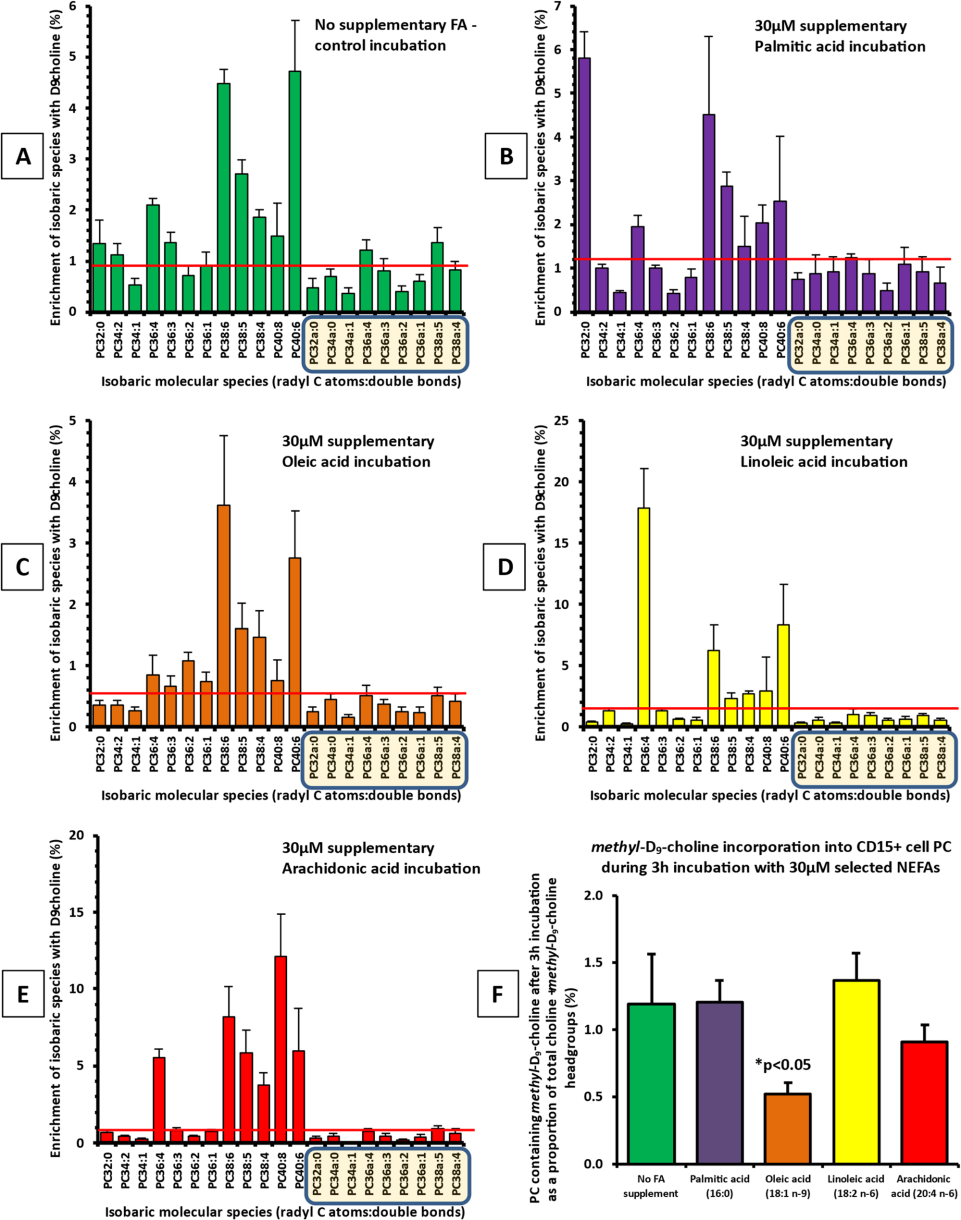
A pictorial overview of molecular phosphatidylcholine distribution of CD15 + cellular *methyl*-D_9_-choline enrichment after 3-hours incubation with selected NEFAs, no supplement (**A**), palmitic acid (**B**), oleic acid (**C**), linoleic acid (**D**), arachidonic acid (**E**), and total *methyl*-D_9_ incorporation for individual NEFAs compared to the controls using student t- test (**F**)

To visualise any differences in incorporation of individual NEFAs into isolated CD15 + cells, we focused on *methy*l-D_9_-choline label enrichments achieved at 3 h. For each donor, we compared PC synthesis patterns in separate incubations of CD15 + cells with individual 30µM albumin-bound PA, OA, LA and AA comparable to those undertaken for FACS analyses. All supplementary FAs resulted in fractional compositions of the newly synthesised *methyl*-D_9_-choline labelled PC that were significantly altered compared with A control cell incubations without added FA (Fig. 6).

The patterns, expressed as a percentage enrichment of the individual and total PC species (Fig. 6, panels A - F) revealed quite distinct differences both in individual species and total enrichment. Importantly, mean values for this deuteriated PC synthesised during the 3 h incubation, whether in control or in FA-supplemented cells, represented less than 1.5% of combined labelled and unlabelled ion intensities, as indicated both by the red dotted line for each supplemented NEFA and in panel F for direct comparison, showing the mean enrichments. These total incorporations (panel F) were lower than those recorded in our previous observations with cultured cells proliferating in vitro [26–28]. Consequently, incorporation rates likely reflect “housekeeping” membrane PC maintenance/turnover and/or functional requirements rather than extensive membrane PC accretion to support proliferation. Interestingly, OA (18:1, *cis-9*) supplementation showed significantly lower enrichment compared with other supplementations (Fig. 6C and F, *p* < 0.05).

### Enrichment of specific PC molecular species

**Fig. 7:**
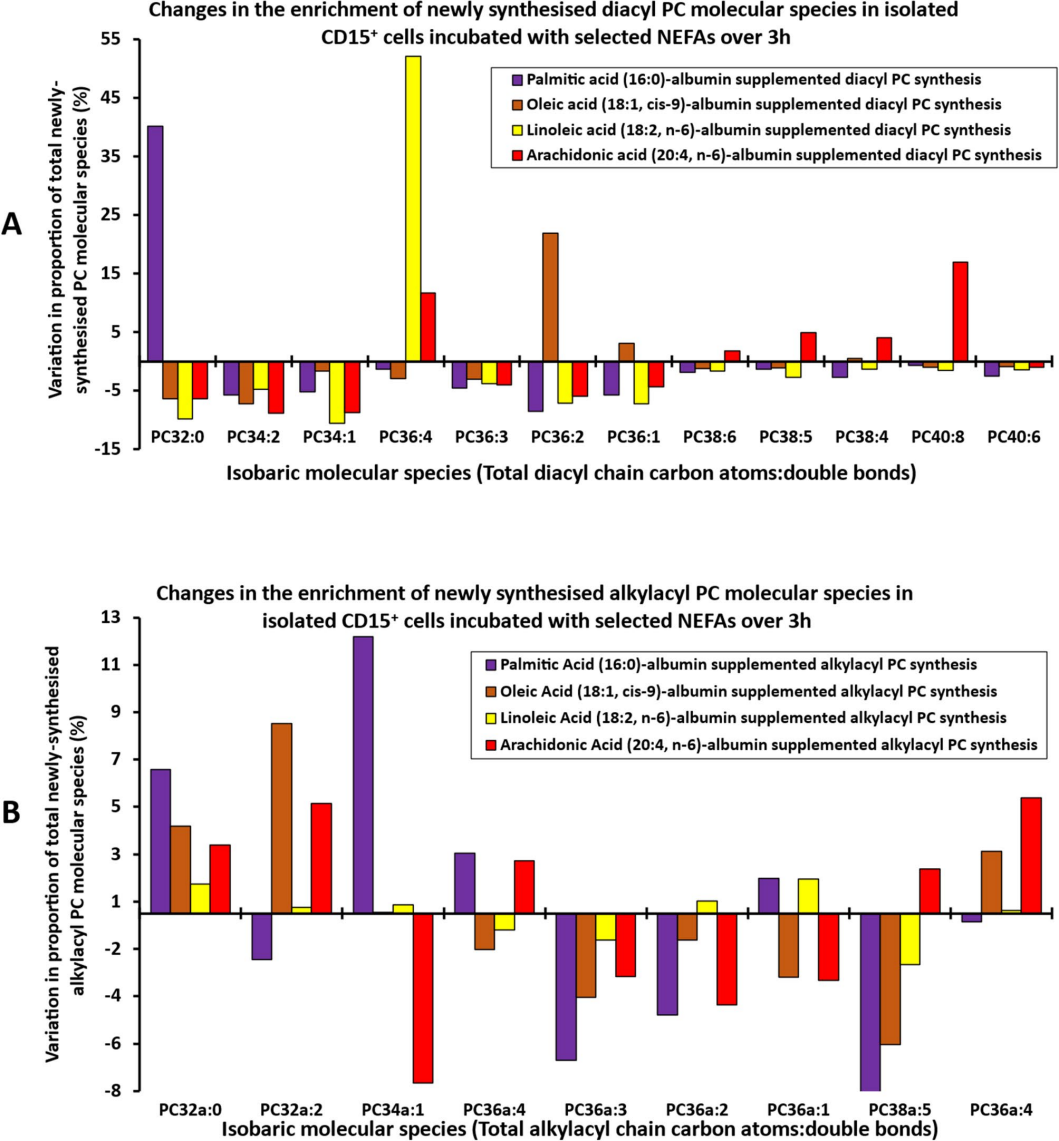
A pictorial overview of the changes in the *methyl*-D_9_-choline enrichment individual molecular fractional phosphatidylcholine distribution of diacyl (**A**) and alkylacyl (**B**) in the CD15 + cells after NEFAs supplementation

An alternative approach to present and understand the effect of NEFA supplementations on the patterns of PC synthesis is through the analysis of change in molecular species synthesised relative to the pattern seen in unsupplemented CD15^+^ cells. In Fig. 7A and B, positive values above the X axis represent the proportional enrichment of diacyl (Fig. 7A) or alkylacyl (Fig. 7B) molecular species synthesised compared with no FA supplement. Negative values represent a fractional depletion in molecular species synthesised.

Among the diacyl species, for PA supplemented cells, PC32:0 identified as predominantly PC16:0/16:0, was enriched at the expense of all other molecular species (Fig. 7A). Similarly, LA supplementation resulted in only one enriched species, PC36:4, which was consistent with PC18:2/18:2. In contrast, OA and AA supplementation was associated with enrichment in two and five isobaric species respectively. For OA, the enrichment in PC36:1 and PC36:2 may indicate acylation of newly synthesised PC at the sn-2 position of PC18:0/18:1 and both sn-1 and sn-2 positions in PC18:1/18:1. The five newly synthesised isobaric species enriched by AA exposure, PC36:4, PC38:4, PC38:5, PC38:6 and PC40:8 were likely all AA containing species with PC40:8 as diarachidonyl PC.

Among the isobaric alkylacyl PC species (Fig. 7B), the apparent enrichments were more complex with some enriched species incapable of having incorporated the relevant supplementary NEFA. Consequently, while presented here for comparison, they may lack the ability to accurately inform. This reflects a combination of the single available acylation opportunity, changes in what are smaller abundance molecular species and a different biosynthetic pathway for alkylacyl PC.

## Discussion

The observation that each of the four supplementary NEFAs, rather than AA alone, was able to induce significant marker expression responses compared with unsupplemented controls was unexpected, although there was some variation in the apparent magnitude of effect. Increased CD11a and CD11b together with a reduction in CD62L, is known to accompany the transition from quiescent, circulating neutrophils to their activated inflammatory immunophenotype as they pass through the vascular endothelium during the recruitment process [15]. Even in the absence of physical passage from circulation to tissue, our data in vitro was consistent with the activation pattern of markers observed clinically in the neutrophils migrating into the lungs of ARDS patients [31].

Exposure to, and uptake of, single molecular species of these saturated, monounsaturated or polyunsaturated NEFAs and incorporation into LB and/or membranes appeared to be sufficient to induce CD15 + cell priming in vitro, with respect to activation markers, rather than specifically requiring transmigration and accompanying AA enrichment. Use of single NEFA incubations alone in vitro clearly differs from exposure to the full spectrum of circulating NEFAs seen in vivo. So, it is possible that, when presented with a choice, a preferential and selective uptake occurs in vivo which might explain why activated alveolar human neutrophils display AA enrichment in ARDS [9, 18]. However, it should be noted that early work with mixed FA supplementations of human neutrophils in vitro did not show selectivity in uptake [19] which may suggest instead an increased bioavailability of AA in ARDS as seen with COVID-19 ARDS [8]. Although we cannot exclude the possibility that the activation state of the control incubations may have been a consequence of trace endotoxin contamination, the robust effects of the fatty acid incubations were additional to any such baseline activation.

Depending upon the extent of FA incorporation into membrane lipids, the range of consequences could induce anything from major biophysical effects across the whole PM to smaller but more targeted effects confined to specific areas or microdomains. Alteration in the acyl composition of human neutrophil membrane phospholipids is known to be regulated in part by four membrane bound acyltransferases, MBOAT1, MBOAT2, MBOAT5 and MBOAT7 [30]. Incubation of neutrophils with supplementary NEFAs in vitro has previously shown uptake and a bi-spatial incorporation into (i) membrane phosphatidylcholine (PC) and (ii) an enlarged pool of lipid bodies (LBs) [9, 18]. While excess FA accumulation into LB neutral lipid is a common cellular response that sequesters potentially lipotoxic NEFAs [32], an AA enrichment in PC molecular species has the potential to alter PM biophysical characteristics; a phenomenon known to affect interactions with membrane-associated proteins [33]. So, in addition to membrane deformability, variation in PM lipid composition also modulates membrane microdomain formation [34] that may in turn influence recruitment of CD protein markers of neutrophil activation.

Whole cell pools of membrane PC in different tissues exhibit specific lipid compositions related to their functions and phenotypes [35]. A tightly defined and regulated specificity is observed for phospholipids, both in class distribution and in individual molecular species compositions. Underpinning this regulation are robust, phenotype-driven homeostatic constraints that operate *in vivo.* These effectively confine the range of permitted changes in membrane lipid compositions, maintaining both their integrity and their biophysical properties consistent with facilitating unique functions of each tissue. Lipid homeostasis, as developing cells and tissues transition to their mature phenotype, is dynamic and responds to their terminal differentiation requirements. In the case of neutrophils, any alterations evaluated in compositional analyses, from dietary manipulation studies in vivo [36, 37], have typically focused on inflammatory mediator output rather than on the dynamics of their membrane composition.

Ample evidence indicates that bulk membrane compositions of both cell lines and cells differentiated in vitro are susceptible to large alteration, but this relies on sustained FA supplementation of culture medium in vitro over prolonged periods [16, 27, 38–44]. Indeed, we have observed substantial change over weeks of growth and subculture of promyelocytic HL-60 cells [16] maintaining cell integrity although not necessarily function. We have attributed this to the progressive loss in vitro of some of the homeostatic regulatory mechanisms normally seen in vivo. Importantly, the short-term culture used here, together with the least partial retention of those same homeostatic mechanisms in freshly isolated primary cells cultured in vitro, likely renders the cells’ permissible range of variation of existing lipid composition much smaller. Consequently, this represents a closer approximation to mechanisms operating in vivo [40].

After 3 h, deuteriated PC, even in proliferating, cultured cells incubated with *methyl*-D_9_-choline, typically only increases total PC by a few percent distributed between multiple molecular species [27]. In this study, the percentages varied but were consistently less than 1.5% This suggests that any biophysical relevance of membrane changes may only be substantial if highly localised, and dependent upon the precise spatial distribution of a small quantity of newly synthesised lipid within the 3 h incubation period. Lipid organisation in neutrophil PMs is known to change during transmigration [45], with temporospatial variations in lipid rafts, labile regions of lipid heterogeneity (membrane microdomains), which in turn suggests that NEFA supplementation here may presage similar changes. In neutrophils, specific functional CD markers are differentially expressed during activation, with lipid raft recruitment [46] of L-selectin (CD62L) during the cell rolling phase [47] prior to transmigration which is then shed as CD11a [48] and CD11b [49] are recruited to lipid rafts during activation. In the case of the ARDS patients, as noted previously, PM display of these activation markers coincides with the whole neutrophil enrichment in AA [9]. It seems likely, on the basis of our data here, that enrichment of the other NEFAs evaluated may also have capacity to effect similar membrane microdomain changes that facilitate surface expression of these activation markers [43, 50, 51].

## Conclusions

In this study, we have characterised and demonstrated CD15 + cell membrane phospholipid composition and phosphatidylcholine flux by the incorporation of *methyl*-D_9_ choline chloride after four NEFA supplementation. All NEFAs alerted the enrichment and newly synthesised membrane PC composition and cell surface activation markers, suggesting potential targets for modulation of neutrophil mediated inflammation.

## Data Availability

The datasets used and/or analysed during the current study are available from the corresponding author on reasonable request.

## Abbreviations

AA: Arachidonic acid
ARDS: Acute respiratory distress syndrome
CD: Cluster differentiation
FA: Fatty acid
FACS: Fluorescence activated cell sorting
LA: Linoleic acid
NEFA: Non esterified fatty acids
OA: Oleic acid
PA: Palmitic acid
PC: Phosphatidylcholine
PUFA: Polyunsaturated fatty acid
